# A Case of Thrombotic Microangiopathy Secondary to Hypertensive Emergency

**DOI:** 10.7759/cureus.24237

**Published:** 2022-04-18

**Authors:** Sai Samyuktha Bandaru, Shiva Charan Anaji, Ifeoluwa T Stowe

**Affiliations:** 1 Internal Medicine, Baton Rouge General Medical Center, Baton Rouge, USA

**Keywords:** hypertension-induced tma, thrombotic thrombocytopenic purpura, ttp, hypertensive emergency, thrombotic microangiopathy (tma)

## Abstract

Hypertension causing thrombotic microangiopathy (TMA) is one of the several etiologies of TMA, which causes endothelial damage and thrombosis of microvasculature, leading to hemolytic anemia, thrombocytopenia, and ischemic organ damage. Renal microvasculature involvement leading to renal dysfunction is most frequently seen in TMA but the degree of dysfunction varies with etiology. If left untreated, TMA carries a risk of high mortality, so it is extremely important for early identification of the cause of TMA. Plasma exchange is a commonly used treatment modality for TMA; however, it is not always necessary. Hypertension-induced TMA can be safely treated with antihypertensives, without the need for invasive plasma exchange. We report a 37-year-old African American hypertensive lady presenting with hypertensive emergency and TMA with rapidly progressing renal dysfunction. The patient had improvement in her platelet count after her blood pressure was reduced in a controlled manner.

## Introduction

Thrombotic microangiopathy (TMA) is a pathological entity caused by a heterogeneous group of diseases and is characterized by abnormalities in the vessel wall of capillaries and arterioles leading to microvascular thrombosis. This results in mechanical hemolysis and platelet consumption and presents as hemolytic anemia, thrombocytopenia, and tissue injury associated most commonly with renal dysfunction [1]. The primary TMA syndromes are thrombotic thrombocytopenic purpura (TTP), Shiga toxin-mediated hemolytic uremic syndrome (HUS), complement-mediated TMA (C-TMA), and drug-induced TMA. Systemic disorders associated with TMA include severe hypertension, systemic lupus erythematosus, pre-eclampsia with severe features, and hemolysis, elevated liver enzyme, low platelet count (HELLP) syndrome, as well as complications following hematopoietic stem cell or solid organ transplantation [2].

Severe hypertension, which is defined as a systolic blood pressure > 180 mmHg and/or a diastolic blood pressure > 120 mmHg, remains an important and preventable cause of TMA [3]. Hypertensive emergencies are characterized by the aforementioned blood pressure values with evidence of acute, ongoing target organ damage, as seen in our patient who had an acute kidney injury (AKI) [4]. The terms “malignant hypertension” and “accelerated hypertension” were commonly used terms in the past for severe hypertension. In our case, the patient was evaluated for other causes of TMA as listed above and after excluding other etiologies, we confirmed the etiology of her TMA was due to severe hypertension.

## Case presentation

A 37-year-old African American female presented to our emergency department with complaints of intractable nausea and non-bilious, non-projectile vomiting for a duration of two weeks; she also had frontal dull headaches. Her medical history was significant for hypertension, which was diagnosed 20 years prior and evaluation for secondary hypertension in the past had been negative. She endorsed a history of long-standing poor blood pressure control due to medication non-compliance and was on amlodipine 10 mg daily and carvedilol 12.5 mg twice daily. Other medical problems included type 2 diabetes mellitus on oral anti-hyperglycemic medication. No other significant surgical or family history was noted. On examination, her blood pressure was 230/142 mmHg without right-left arm variation and tachycardic with a pulse rate of 132/min, a respiratory rate of 18 breaths per minute, and was afebrile. She had clear breath sounds, her heart rate was regular without any murmurs, and the neurologic examination was normal. The rest of the physical examination was unremarkable.

Further investigation revealed elevated blood urea nitrogen (BUN) of 49 mg/dl, creatinine of 6.74 mg/dl, hemolytic anemia with hemoglobin of 7.4 g/dl, haptoglobin < 8 g/dl, lactate dehydrogenase (LDH) of 721 IU/L, and total bilirubin of 1.9 mg/dl with normal liver enzymes. Coombs test was negative and the peripheral smear showed schistocytes, suggestive of microangiopathic hemolytic anemia. Furthermore, she was thrombocytopenic with a platelet count of 33,000 per microliter. As she had a triad of features concerning TMA, there was a high suspicion of hypertension-induced TMA due to severe hypertension at the time of presentation; however, further workup was done to identify etiology. The PLASMIC score was calculated while awaiting ADAMTS (a disintegrin-like and metalloproteinase with thrombospondin type 1 motif) activity results. The PLASMIC score was 4, i.e., low probability for TTP [5], and ADAMTS activity was normal. Additional workups with blood, urine cultures, hepatitis B and C, and HIV were negative, and complement levels were unremarkable. Renal ultrasound was done to rule out any other etiologies for renal dysfunction; renal ultrasound was negative for any masses, stones, or obstructive etiology. Serum protein and urine protein electrophoresis were unremarkable for monoclonal paraproteinemia.

After excluding all possible etiologies, our patient was diagnosed with hypertension-induced TMA. She was initiated on intravenous labetalol for management of hypertensive emergency in a controlled manner (with a target reduction in her systolic blood pressure of not greater than 25% in the first 24 hours). She was transitioned to oral antihypertensive medications with a target systolic blood pressure of not less than 150 mmHg. Hence, the patient's blood pressure slowly improved in a timely manner. She required one unit of packed red blood cell transfusion on day five of the hospital course; however, her platelets improved without any transfusion and LDH trended down with controlling her blood pressure (Table 1). Her creatinine remained stable (ranging from 6.6 to 7 mg/dl), and she was evaluated by a nephrologist since she had no uremic symptoms and continued to make adequate urine throughout the hospital course; she did not require initiation of dialysis. She was subsequently discharged and was closely followed by the nephrology team. Her blood pressure was well controlled during her follow-up visit.

**Table 1:** Chronological assessment of lab values showing improvement in platelets and downtrending lactate dehydrogenase (LDH) after initiation of antihypertensive therapy.

Day of disease	Hemoglobin	Platelets (x10^9^/L)	Blood urea (mg/dl)	Serum creatinine (mg/dl)	LDH
Day 1	7.4	33	49	6.74	721
Day 2	6.9	33	49	6.63	697
Day 3	7.0	49	60	6.89	640
Day 4	7.0	70	60	7.13	538
Day 5	6.6	112	65	7.44	
Day 6	7.5	162	66	7.61	

## Discussion

TMA has various etiologies and is diagnostically challenging. Hypertensive TMA is one of the several etiologies of TMA causing thrombosis of microvasculature leading to fragmentation of red blood cells causing microangiopathic hemolytic anemia, thrombocytopenia, and ischemic end-organ damage [6]. Involvement of renal microvasculature causing renal dysfunction is commonly associated with TMAs. Primary etiologies are TTP, Shiga toxin-mediated HUS, and complement-mediated atypical HUS [7]. It is extremely important to differentiate between different etiologies, as management depends on the type of TMA.

Different etiologies of TMAs can be excluded based on their presentations and diagnostic workup. All the TMAs are commonly associated with a triad of features with hemolytic anemia with schistocytes on peripheral smear, thrombocytopenia, and end-organ damage. TTP can have characteristic findings of fever, altered mental status, and renal dysfunction; if it is due to acquired ADAMTS-13 deficiency, ADAMTS-13 levels are diagnostic for TTP. Shiga-meditated HUS patients usually present with diarrhea and Shiga toxin levels are diagnostic in these patient populations. Shiga-meditated HUS can be managed with supportive care. A complement factor workup can be done to rule out C-TMA. C-TMAs are treated with anti-complement therapy like eculizumab [8]. Other secondary causes of TMA can be ruled out by obtaining blood cultures, hepatitis, HIV levels, and antinuclear antibody (ANA) levels.

TTP and hypertensive TMA share many similarities; hence, it is important to differentiate them, as emergent plasmapheresis is required for TTP whereas severe hypertension-induced TMA can be managed by effective blood pressure control with antihypertensive medications [9]. Medical history of uncontrolled hypertension, severe hypertension during the presentation, and ruling out other etiologies are helpful in the diagnosis of hypertensive TMA. Lack of ADAMTS-13 deficiency can differentiate it further away from TTP. Although plasma exchange is lifesaving, unnecessary therapy should be avoided as it is an expensive therapy and associated with complications including bloodstream infection and allergic reactions [4,10]. The PLASMIC score is a helpful tool, which helps with prompt diagnosis of TTP while awaiting ADAMTS-13 activity results. PLASMIC score includes platelet count < 30,000/109/L, hemolysis with either increased reticulocytes, increased LDH, decreased haptoglobin, or increased indirect bilirubin, no active cancer, no history of solid organ or stem cell transplant, mean corpuscular volume (MCV) < 90 fl, international normalized ratio (INR) < 1.5, and creatinine < 2.0 mg/dl, with one point for each positive finding. PLASMIC score greater than 6 is highly predictive of TTP and a score less than 5 is a low risk for TTP [5]. Unlike TTP, patients with TMA secondary to severe hypertension respond well to antihypertensive medications and do not require plasma exchange but may, unfortunately, progress to persistent renal failure [11].

In our case, our patient's etiology of hypertension-induced TMA was confirmed with her history of uncontrolled blood pressure with a severely elevated blood pressure of 230/142 mmHg at presentation; she had a classic triad of thrombocytopenia, hemolytic anemia, and renal dysfunction, with a low PLASMIC score of 4; she had normal ADAMTS-13 activity and complement levels; and workup for secondary etiologies of TMAs, i.e., HIV, hepatitis, paraproteinemia, and blood and urine cultures, was unrevealing. We treated her with antihypertensives in a controlled manner and her platelet count improved without requiring plasma exchange. The patient's kidney function remained stable and did not require initiation of hemodialysis. She was discharged and was closely followed by nephrologists for monitoring her kidney function.

## Conclusions

TMAs have various etiologies, and it is extremely important for the early identification of the cause of TMA. As TTP is a life-threatening hematological condition, it requires immediate treatment with plasma exchange; however, hypertension-induced TMA does not necessarily require plasma exchange. Due to the rarity of hypertension-induced TMA, it poses a diagnostic challenge to clinicians. Prompt diagnosis of hypertension-induced TMA and initiation of antihypertensive therapy in a timely manner have a favorable prognosis.

## References

[1] Benz K, Amann K (2010). Thrombotic microangiopathy: new insights. Curr Opin Nephrol Hypertens.

[2] Kappler S, Ronan-Bentle S, Graham A (2017). Thrombotic microangiopathies (TTP, HUS, HELLP). Hematol Oncol Clin North Am.

[3] Peixoto AJ (2019). Acute severe hypertension. N Engl J Med.

[4] Thind G, Kailasam K (2017). Malignant hypertension as a rare cause of thrombotic microangiopathy. BMJ Case Rep.

[5] Oliveira DS, Lima TG, Benevides FL, Barbosa SA, Oliveira MA, Boris NP, Silva HF (2019). Plasmic score applicability for the diagnosis of thrombotic microangiopathy associated with ADAMTS13-acquired deficiency in a developing country. Hematol Transfus Cell Ther.

[6] Brain MC, Dacie JV, Hourihane DO (1962). Microangiopathic haemolytic anaemia: the possible role of vascular lesions in pathogenesis. Br J Haematol.

[7] George JN, Nester CM (2014). Syndromes of thrombotic microangiopathy. N Engl J Med.

[8] Nürnberger J, Philipp T, Witzke O (2009). Eculizumab for atypical hemolytic-uremic syndrome. N Engl J Med.

[9] Shibagaki Y, Fujita T (2005). Thrombotic microangiopathy in malignant hypertension and hemolytic uremic syndrome (HUS)/thrombotic thrombocytopenic purpura (TTP): can we differentiate one from the other?. Hypertens Res.

[10] Khanal N, Dahal S, Upadhyay S, Bhatt VR, Bierman PJ (2015). Differentiating malignant hypertension-induced thrombotic microangiopathy from thrombotic thrombocytopenic purpura. Ther Adv Hematol.

[11] Asano T, Mori H (2022). Thrombotic microangiopathy due to malignant hypertension treated exclusively with antihypertensive therapy. Cureus.

